# Supplementary material for: Identification of structures for ion channel kinetic models
Source: PLoS Comput Biol. 2021 Aug 16;17(8):e1008932. doi: 10.1371/journal.pcbi.1008932 (PMC8389848; doi:10.1371/journal.pcbi.1008932)
Supplement: S1 Appendix — (DOCX) [file pcbi.1008932.s001.docx]

Identification of Structures for Ion Channel Kinetic Models

Kathryn E. Mangold^1^, Wei Wang^2^, Eric K. Johnson^2^, Druv Bhagavan^1^, Jonathan D. Moreno^1,2^, Jeanne M. Nerbonne^2,3^, and Jonathan R. Silva^1*^

^1^Department of Biomedical Engineering, Washington University in St. Louis, St. Louis, Missouri, United States of America

^2^Department of Medicine, Cardiovascular Division, Washington University School of Medicine, St. Louis Missouri, United States of America

^3^Department of Developmental Biology, Washington University School of Medicine, St. Louis, Missouri, United States of America

*Corresponding author

Email: [jonsilva@wustl.edu](mailto:jonsilva@wustl.edu) (JRS)

**Equations Illustrated in S2 Fig**

Generalization loss quantifies how much larger the current validation cost is compared to the minimum validation cost seen across all iterations seen so far:

$$\begin{aligned} Generlization Loss \left( GL \right)=\frac{C_{val}\left( t \right)}{{minC}_{val}} -1\#\left( 1 \right) \end{aligned}$$

Progress quantifies how much the average training cost is larger than the minimum cost seen in last *k* optimization iterations**:**

$$\begin{aligned} Progress \left( P \right)=1000*\frac{\sum_{t^{'}=t-k+1}^{t} C_{tr}\left( t^{'} \right)}{k*\min C_{tr}\left( t^{'} \right)}-1\#\left( 2 \right) \end{aligned}$$

The ratio of generalization loss to progress, *Q*, determines when the optimization should be terminated:

$$\begin{aligned} Q=\frac{GL}{P}\#\left( 3 \right) \end{aligned}$$

These equations are adapted from [1].

**Methods for S6-S8 Figs**

***I_Na_ training set of voltage-clamp protocols:***

Steady state activation: Steady-state probabilities were found at -120 mV. For the voltages between -80 and 25 mV for the sodium channel, the peak open probability (a measure of peak current) was recorded at each voltage for 10 ms during the step depolarization and normalized to the max.

Steady state inactivation: Steady-state probabilities were found at -120 mV. Each preliminary voltage step between -105 and -20 mV was held for 200 ms. The peak open probability (a measure of peak current) was then recorded after a test pulse at -20 mV for 20 ms and normalized to the max.

Recovery from Inactivation: Steady-state probabilities were found at -120 mV. A depolarizing pulse of -20 mV for 20 ms was applied followed by a hyperpolarizing pulse of -120 mV of variable time intervals (1-75 ms). Peak current was then recorded and normalized after a pulse at -20 mV for 20 ms

Normalized Current Traces: Steady-state probabilities were found at -120 mV. Following a step depolarization to -10 mV, and 10 mV for 1.4 ms, the normalized current was recorded at intervals of 0.1 ms.

*Maximum Open Probability:* To best match the original sodium simulated current, and thus eliminating the need for rescaling conductance for each individual model, maximum open probabilities of 0.27, 0.31, 0.29 at -10, 0, 10 mV, respectively (calculated from ten Tusscher 2006 solved in MATLAB with ode15s) were enforced.

***I_Na_ validation set of voltage-clamp protocols:***

Additional data points were extrapolated from fits to steady state activation, steady state inactivation, and recovery from inactivation. These points were extrapolated at the beginning, middle, and end of simulation intervals (in mV or ms). For example, the steady state inactivation protocol in the I_Na_ dataset examines the interval from -105 mV to -20 mV. Extrapolated validation points were then chosen at -115 mV (extrapolated value of 1), -10 mV (~0) to ensure appropriate channel inactivation outside of the training voltage range. A “midpoint” validation point at -77 mV (extrapolated value of ~0.5) ensures the trajectory of the inactivation curve maintains its trajectory in between explicit data training points. A complete current trace at 0 mV was also used for validation with the same protocol as the other normalized current traces.

**Example Model Structures Rate Parameters:**

Below are the parameters for the example models for the I_to,f_ (S4 Fig) and I_Na_ (S8 Fig) datasets:

Please see Menon et al. [2] and Teed et al. [3] for background on the rate constant derivations. As described in Menon et al., rate constants are guaranteed to satisfy microscopic reversibility.

$$\begin{aligned} r_{\mathrm{ij}}=\exp\left( a+b*\tanh\left( \frac{v+args_{1}}{args_{2}} \right) \right) \end{aligned}$$

**I_to,f_:**

|  | **a** | **b** |
| --- | --- | --- |
| r_31_ | 2.41 | 10.34 |
| r_13_ | 2.06 | 4.02 |
| r_41_ | -6.21 | -3.90 |
| r_14_ | -3.16 | 2.66 |
| r_42_ | -4.35 | 3.45 |
| r_24_ | -5.85 | 6.61 |

args_1_ = 2.78

args_2_= 32.00

**I_Na_:**

|  | **a** | **b** |
| --- | --- | --- |
| r_41_ | -6.60 | 8.04 |
| r_14_ | -1.55 | -1.27 |
| r_51_ | -2.75 | 1.06 |
| r_15_ | -6.23 | -4.62 |
| r_42_ | -1.04 | 11.10 |
| r_24_ | -4.44 | 5.86 |
| r_52_ | -0.54 | 12.05 |
| r_25_ | -12.47 | 10.44 |
| r_53_ | 17.74 | -8.15 |
| r_35_ | 17.29 | -1.00 |

args_1_ = 53.51

args_2_= 29.40

**References**

1. Prechelt L. Early stopping-but when? Neural Networks: Tricks of the trade. Springer; 1998. pp. 55–69.

2. Menon V, Spruston N, Kath WL. A state-mutating genetic algorithm to design ion-channel models. Proc Natl Acad Sci. 2009;106: 16829–16834. doi:10.1073/pnas.0903766106

3. Teed ZR, Silva JR. A computationally efficient algorithm for fitting ion channel parameters. MethodsX. 2016;3: 577–588. doi:10.1016/j.mex.2016.11.001
