# Supplementary figures and images for: Identification of structures for ion channel kinetic models
Source: PLoS Comput Biol. 2021 Aug 16;17(8):e1008932. doi: 10.1371/journal.pcbi.1008932 (PMC8389848; doi:10.1371/journal.pcbi.1008932)

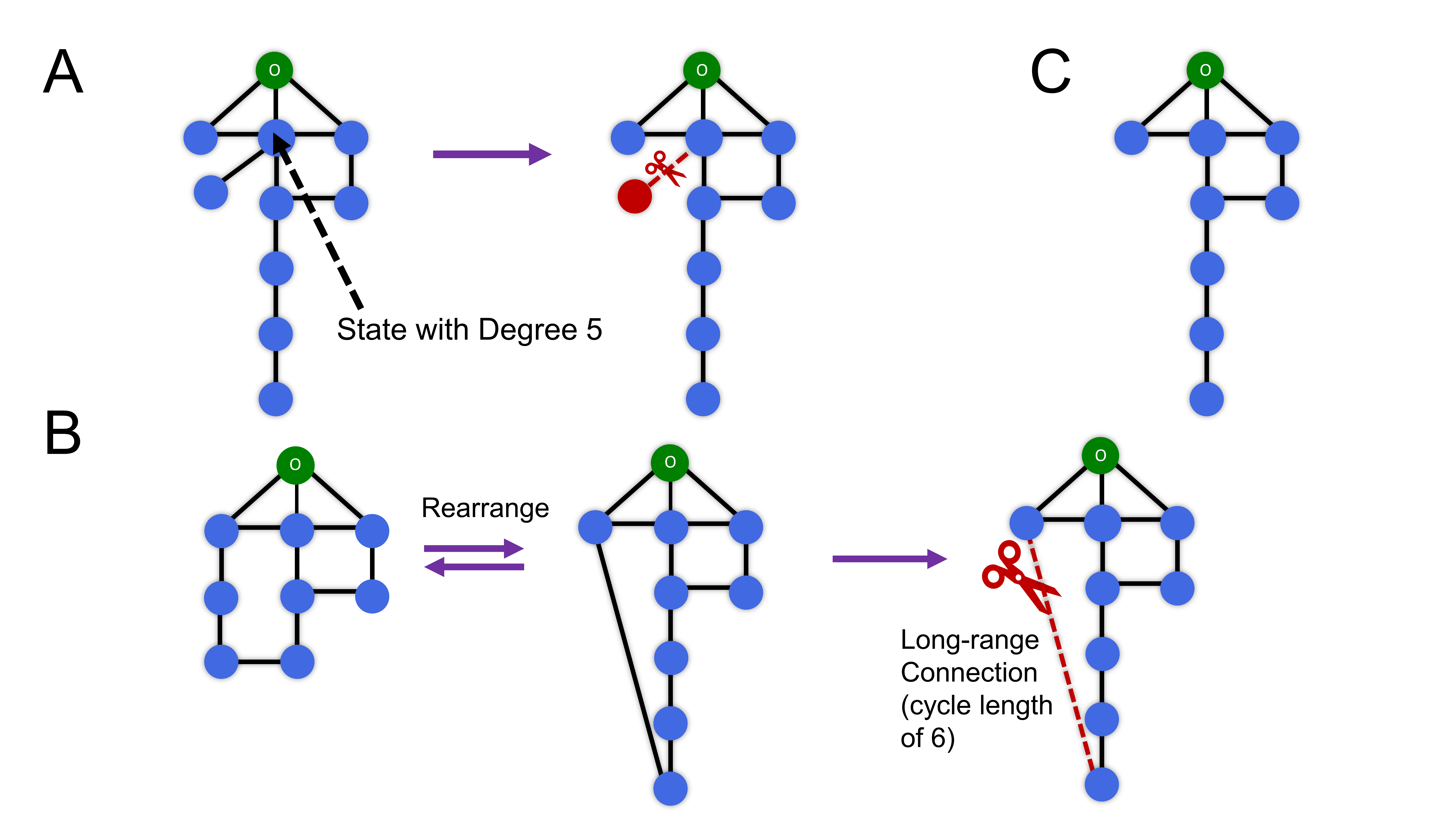

Supplement: S1 Fig — Green states indicate open states while blue indicates all other states A) A representative topology with a labeled state that has five possible connections to other states. By cutting out the red portion (containing the extraneous state and connection), the indicated state then has a degree of four. B) A representative model graph that has cycle length of six. Removal of the indicated red dashed connection breaks the cycle. The resulting topology has a maximum cycle length of four C) A representative 8-state topology that meets the maximum state and long-range connections restrictions illustrated in A & B and is included in the final count in Fig 1E (i.e. is an example of the 72,489 unique rooted topologies). (TIF) [file pcbi.1008932.s002.tif]

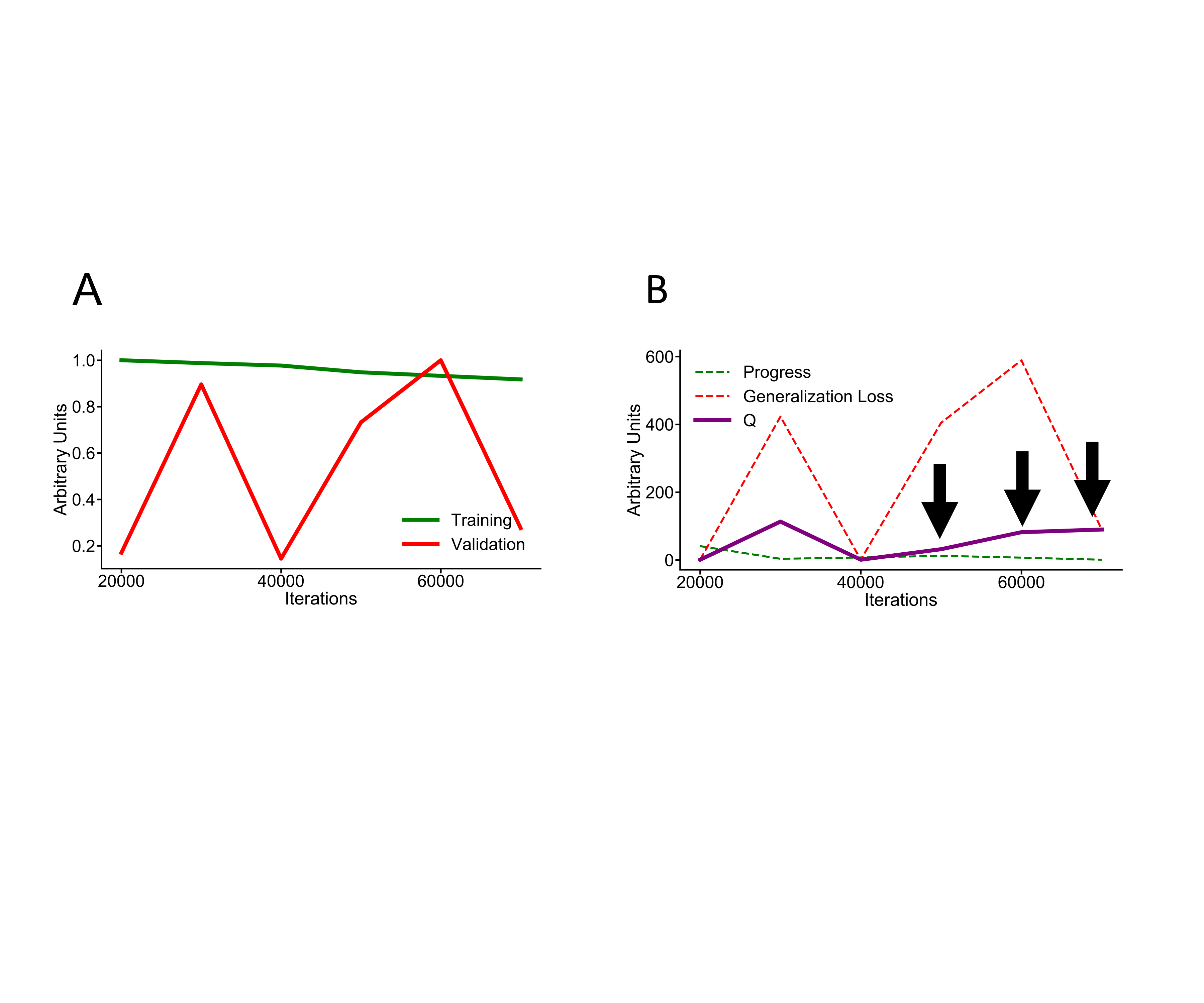

Supplement: S2 Fig — A) Sample normalized training and validation costs during towards the end of an optimization. The training is slow, but steadily declining, while the validation cost is changing erratically at various optimization epochs. B) Measures of progress, generalization loss, and their ratio, Q, over the optimization time period as in A. Progress quantifies how much the average training cost is larger than the minimum cost seen in last k optimization iterations. Generalization loss quantifies how much larger the current validation cost is compared to the minimum validation cost seen across all iterations seen so far. In the example shown, progress (green dashes) stays relatively steady at various epochs reflecting the slow steady decline in training cost. Generalization loss widely fluctuates along with the validation error (red dashes). The ratio of the progress and generalization loss, Q (purple), steadily increases three simulation epochs in a row (as indicated by the black arrows), which signals that early stopping should occur. (TIF) [file pcbi.1008932.s003.tif]

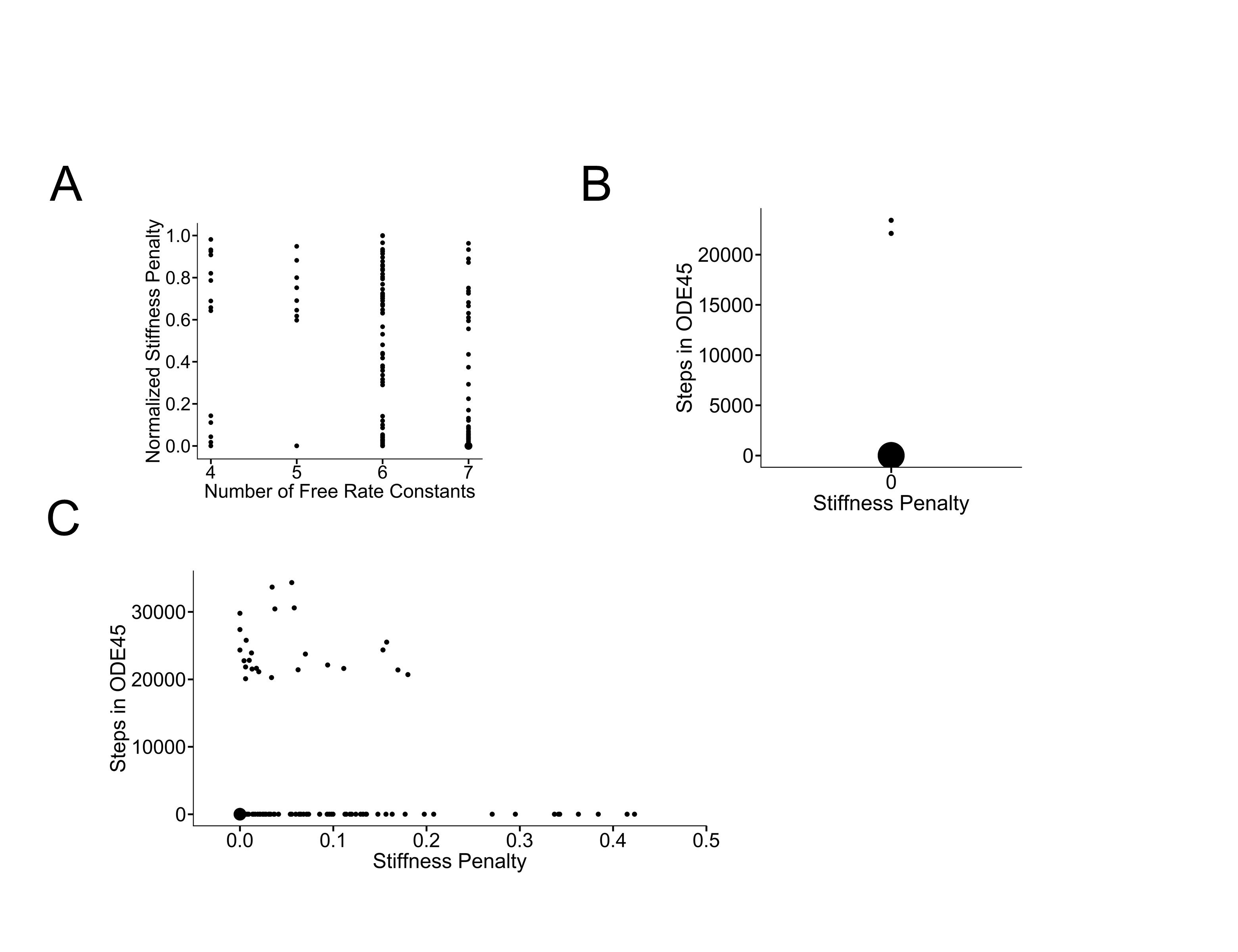

Supplement: S3 Fig — A) The normalized stiffness penalty seen as across all starts as a function of increasing free rate constants. B) The number of steps in an explicit ODE solver (MATLAB’s ODE45) when the penalties are not part of a model’s cost. Only two models may be successfully solved with the less computationally intensive explicit solvers, which indicates the model solutions are inherently stiff. C) When including a measure of model stiffness in the optimization routine [48], more models can successfully be solved in the ODE45 routine. (TIF) [file pcbi.1008932.s004.tif]

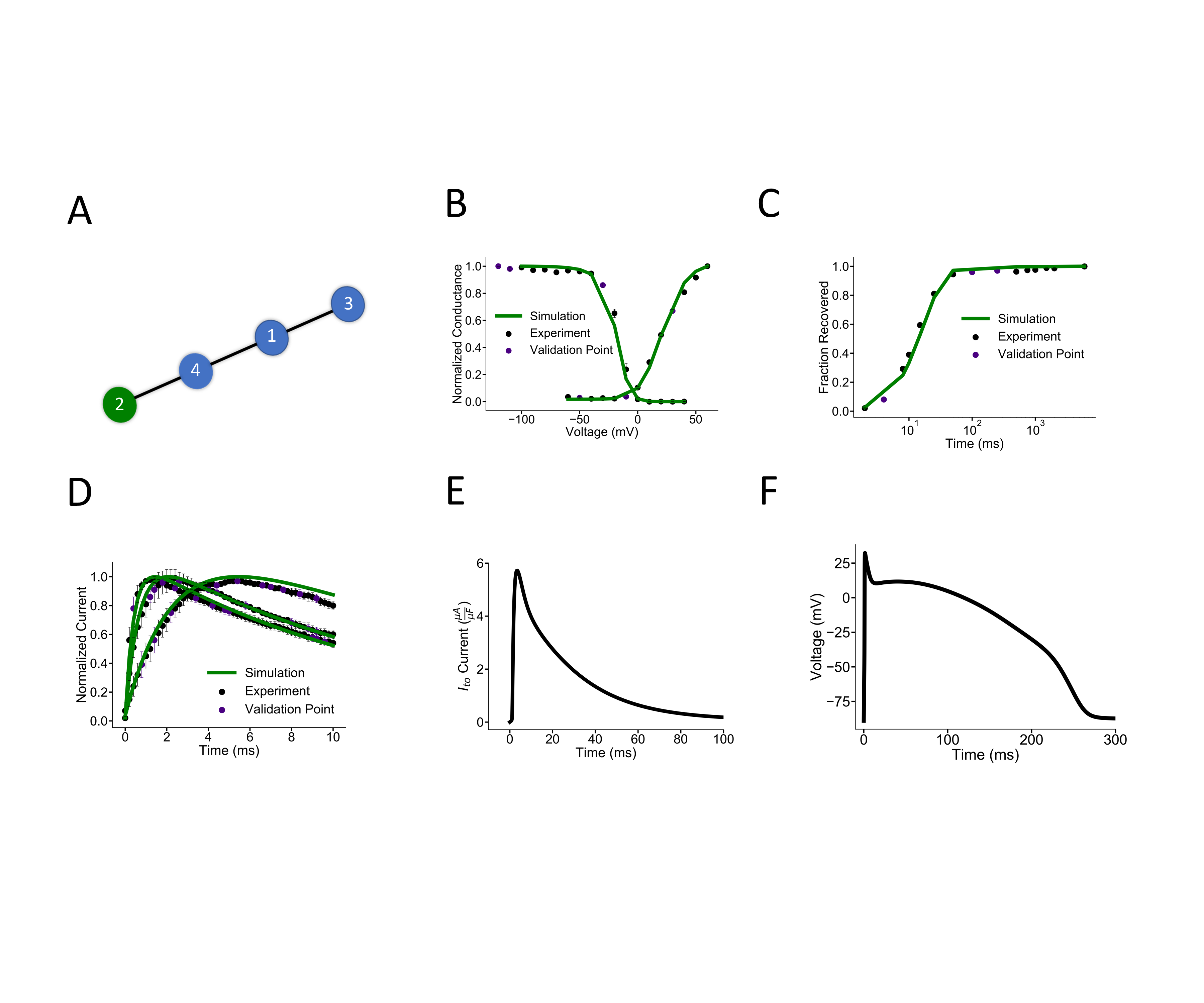

Supplement: S4 Fig — A) Topology of the model B) Experimental data points and simulated fits for conductance voltage and steady state inactivation protocols. Purple validation points indicate points added in the simulation to calculate a model’s validation error when checking for overfitting. C) Experimental recovery from inactivation and simulated fit with added validation points to compute validation error for overfitting prevention. D) Normalized experimental current traces at 20 mV and 60 mV (black) with corresponding simulated traces. In purple is the experimental 40 mV normalized current trace along with its associated fit for validation. E) Ito,f current trace when included in the Tor-Ord [50] human ventricular action potential model (F) All experimental data are from Johnson et al. [60] Experimental data points (black) are mean ± SEM. (TIF) [file pcbi.1008932.s005.tif]

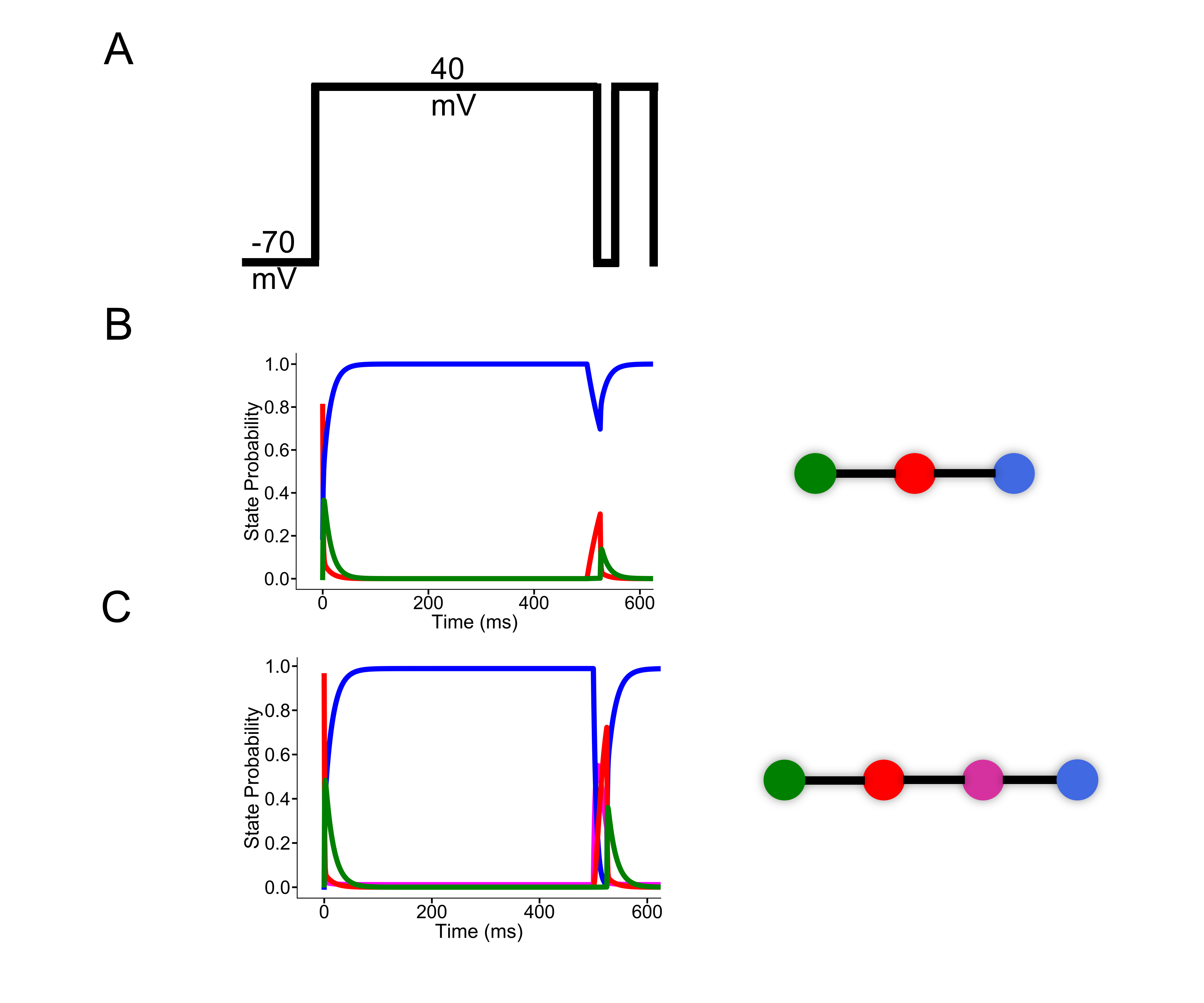

Supplement: S5 Fig — A) Recovery from inactivation protocol. B) State probabilities tracked as a function time throughout the above protocol. The 3-state model shown is representative of unacceptable Ito,f models where recovery from inactivation is slow. Green designates the rooted open state while blue and red indicate the functional hypothetical inactivated state and closed state, respectively. At the resting state (-70 mV), the red “closed” state carries about 80% of the resting state probability while the blue “inactivated” state holds the other 20%. This closed state probability spread at the holding potential essentially “locks in” the voltage-dependent rates at -70 mV as slow. After the depolarizing pulse to +40 mV for 500 ms, the “blue” inactivated state carries 99% of the probability. The 25 ms hyperpolarizing pulse to -70 mV attempts to send the state probability back to the “red” closed state. However, the rate from the blue to red state at -70 mV is quite slow and, on reapplying the depolarizing step, the open state probability is lower than expected after 25 ms of recovery. C) An analogous representation of state probabilities for a 4-state model representative of the acceptable Ito,f models where recovery from inactivation is appropriately fast. State colors are as before with the addition of 4th purple state. At the resting potential, the hypothetical closed state holds 99% of state probability. Therefore, the other states and rates are not “locked in” to slower rates at the holding potential. After the long depolarizing pulse, most state probability is again in the blue inactivated state. In this model, however, the rate from the inactivated state back to closed at -70 mV is fast enough to allow for sufficient recovery from inactivation after 25 ms hyperpolarization (77%). The pink state thus serves as a transitory state that distances the functionally inactive (blue) state from the open state (green). (TIF) [file pcbi.1008932.s006.tif]

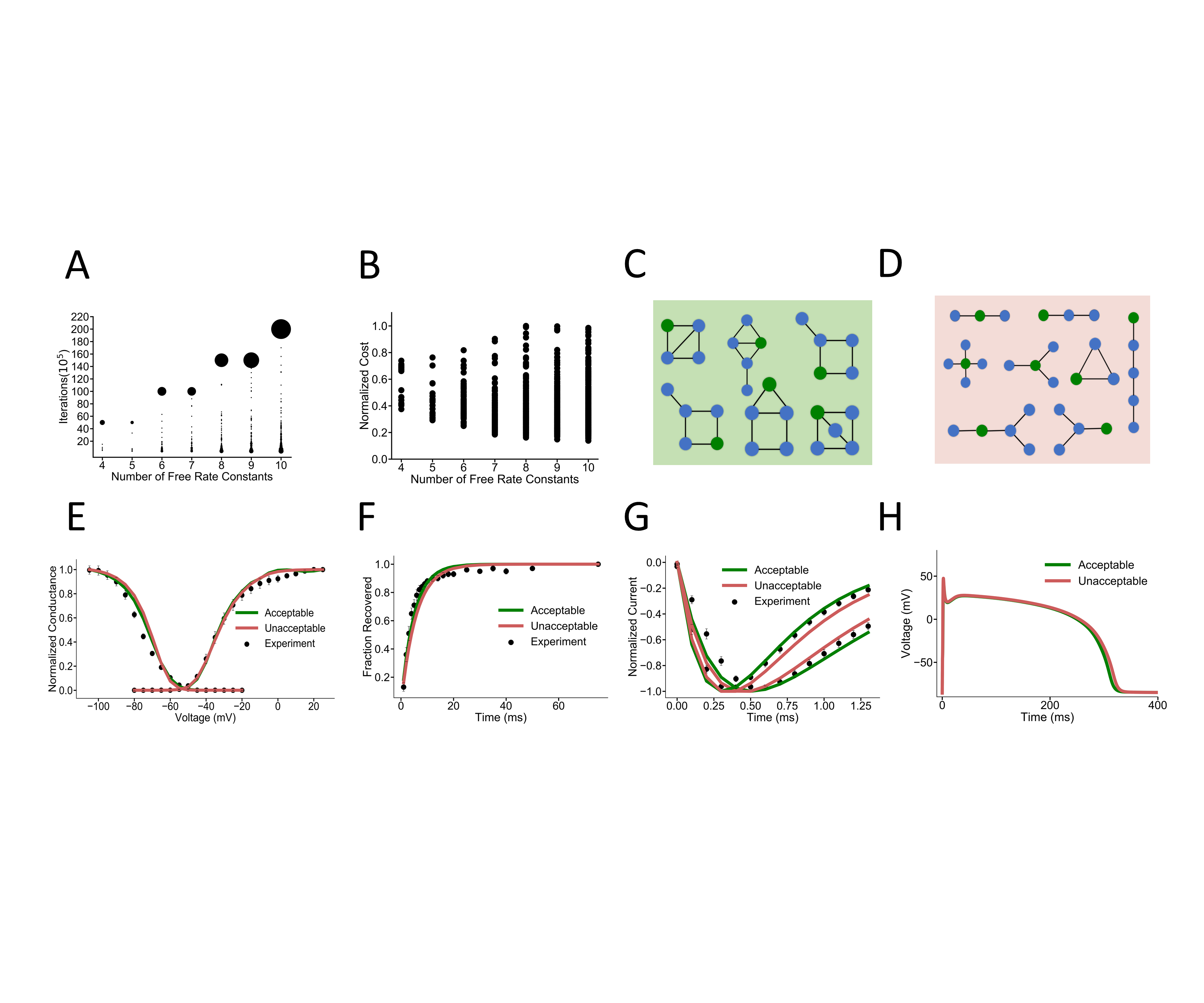

Supplement: S6 Fig — A) Iterations completed for multiple optimizations starts of the models trained on the INa dataset. Most optimization runs complete the maximum number of iterations allowed for the number of free rate constants, but as complexity increases, an optimization is more likely to be stopped early because of potential overfitting. B) Distribution of minimum normalized costs for each model with multiple starts after completing optimization iterations as depicted in A. C) Representative acceptable models for INa. As defined in the text, acceptable models have minimum costs no larger than 300% of the absolute minimum cost. All models have at least 8 free rate constants. D) Representative unacceptable models. All models have minimal states and/or sparsely connected with a range of free rate constants. E) -G) Representative voltage-clamp models fits for steady state activation, inactivation, recovery from inactivation and current traces for models in the acceptable and unacceptable model categories. H) Representative ten Tusscher human ventricular action potentials [52] with replaced modeled acceptable and unacceptable INa currents. (TIF) [file pcbi.1008932.s007.tif]

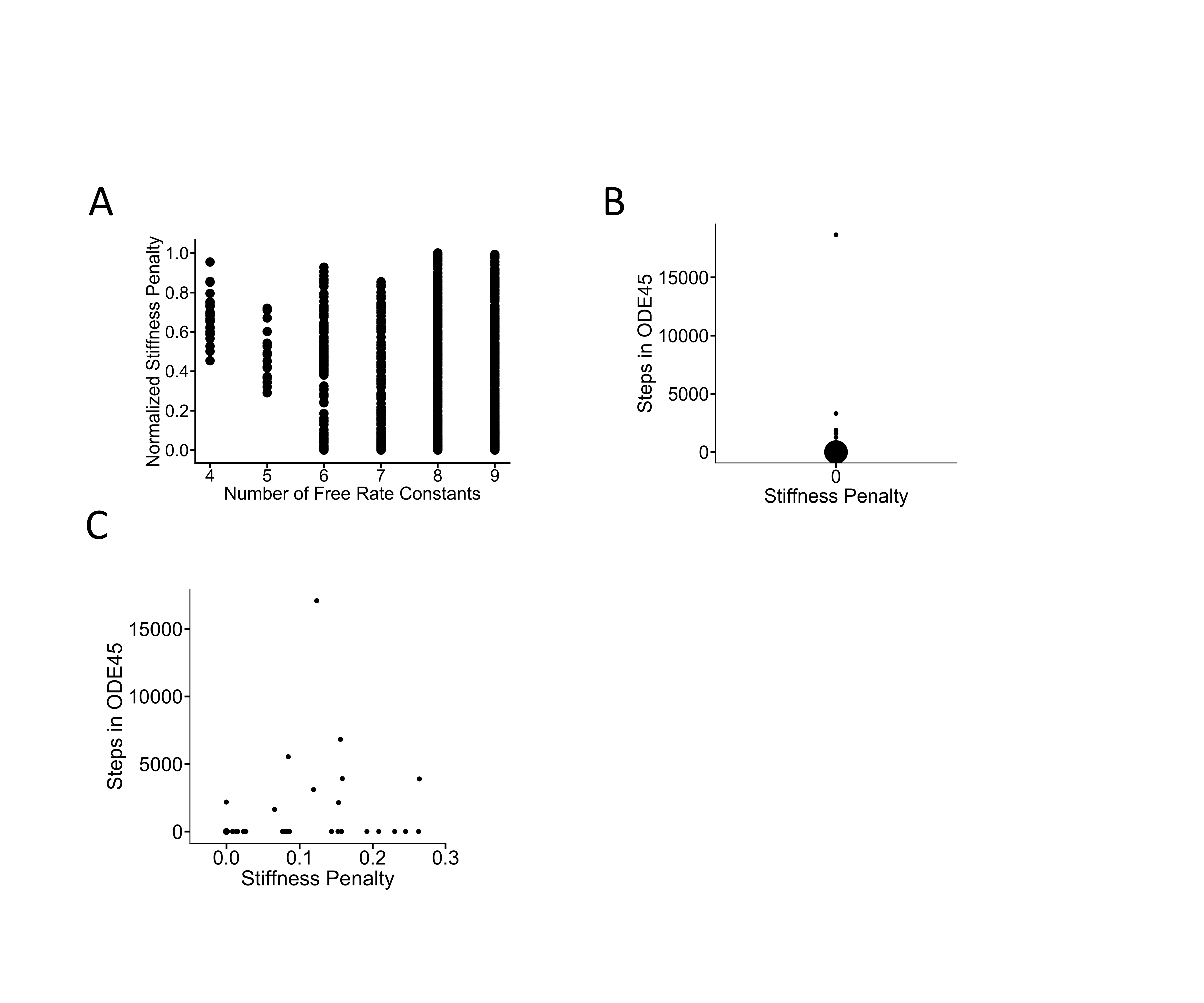

Supplement: S7 Fig — A) The normalized stiffness penalty seen as across all starts as a function of increasing free rate constants. There is decline in the minimum stiffness penalty from four to six free rate constants followed by a plateau in the minimum penalty for topologies with greater than six free rate constants B) The number of steps in an explicit ODE solver (MATLAB’s ODE45) when the penalties are not part of model cost. Few models may be successfully solved with the less computationally intensive explicit solvers, which indicates the model solutions are inherently stiff. C) When including a measure of model stiffness in the optimization routine, more models can successfully be solved in the ODE45 routine. (TIF) [file pcbi.1008932.s008.tif]

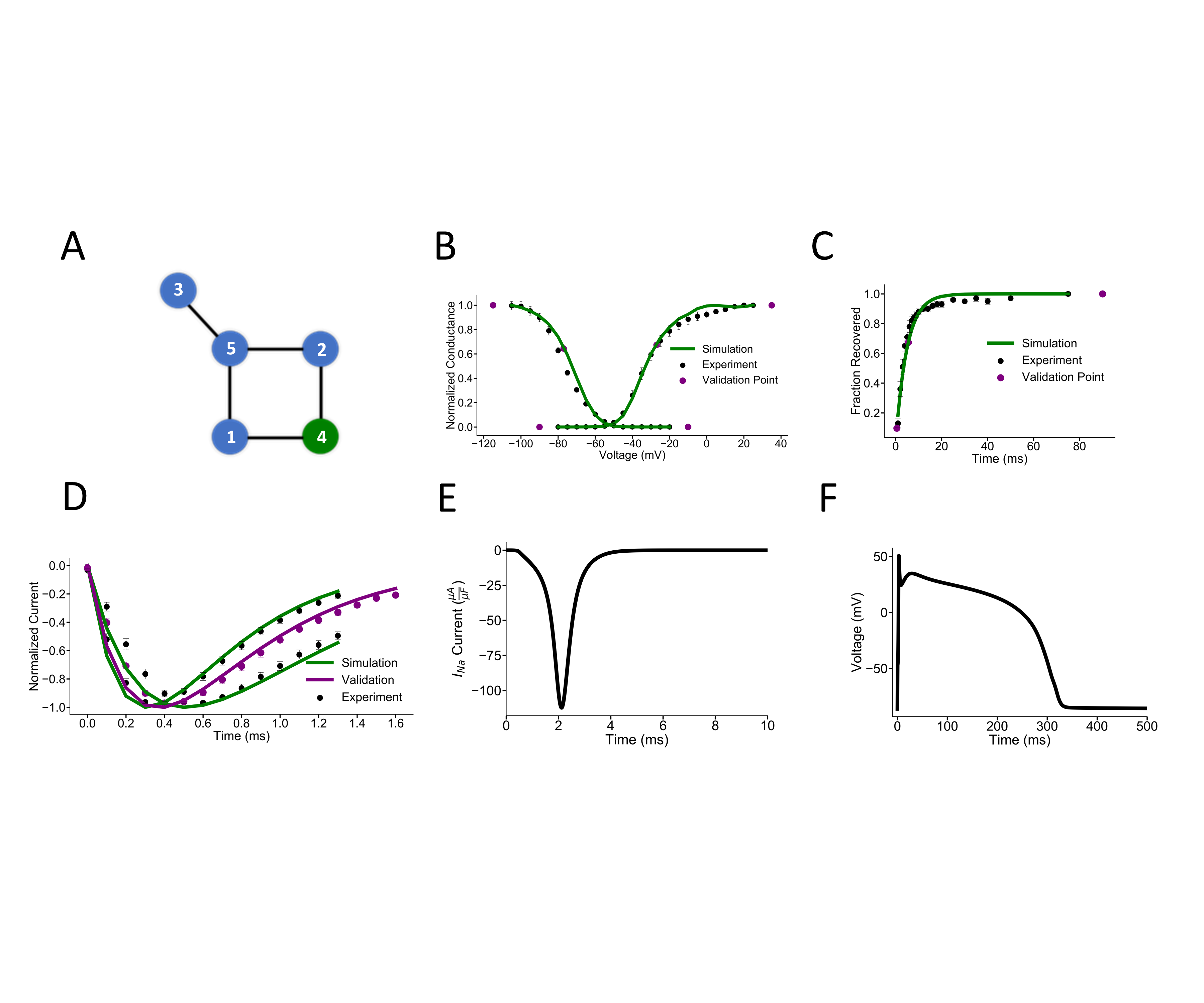

Supplement: S8 Fig — A) Topology of the model and B) Experimental data points and simulated fits for conductance voltage and steady state inactivation protocols. Purple validation points indicate points added in the simulation to calculate a model’s validation error when checking for overfitting. C) Experimental recovery from inactivation and simulated fit with added validation points to compute validation error for overfitting prevention. D) Normalized experimental current amplitudes recorded at -10 mV and 10 mV, plotted as points (black) with corresponding simulated traces in green. The normalized experimental current data acquired at 0 mV along with its associated fit for validation is shown in purple. E) Modeled INa current when inserted into the ten Tusscher [52] human ventricular action potential model and the resulting action potential F). Experimental data points (in black) in B), C) and D) are means ± SEM. (TIF) [file pcbi.1008932.s009.tif]

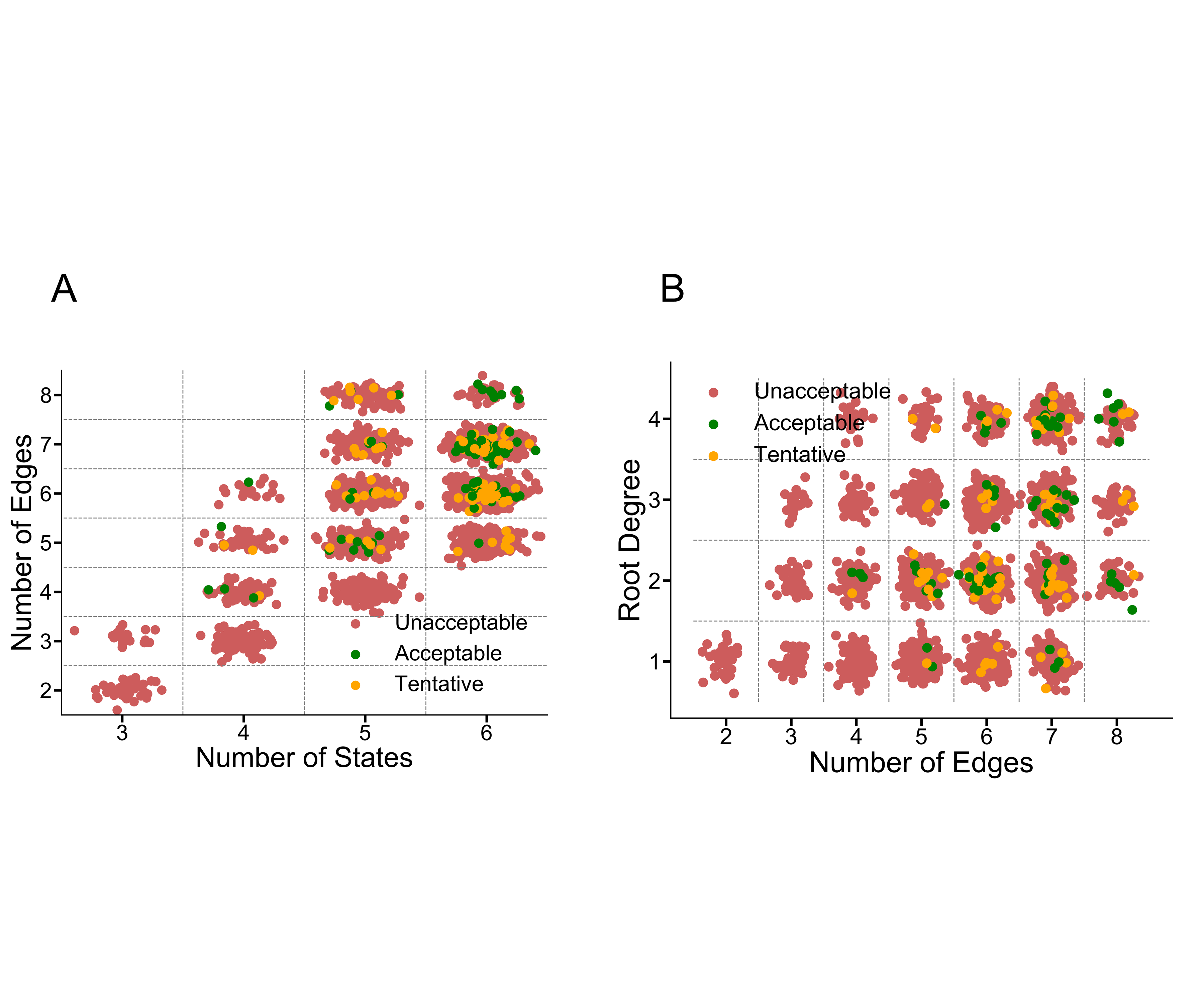

Supplement: S9 Fig — Unacceptable and acceptable labels are as previously defined. Tentative yellow topologies produced acceptable voltage protocol fits but did not perform like other acceptable models in the action potential validation (different degrees of repolarization failure). (TIF) [file pcbi.1008932.s010.tif]

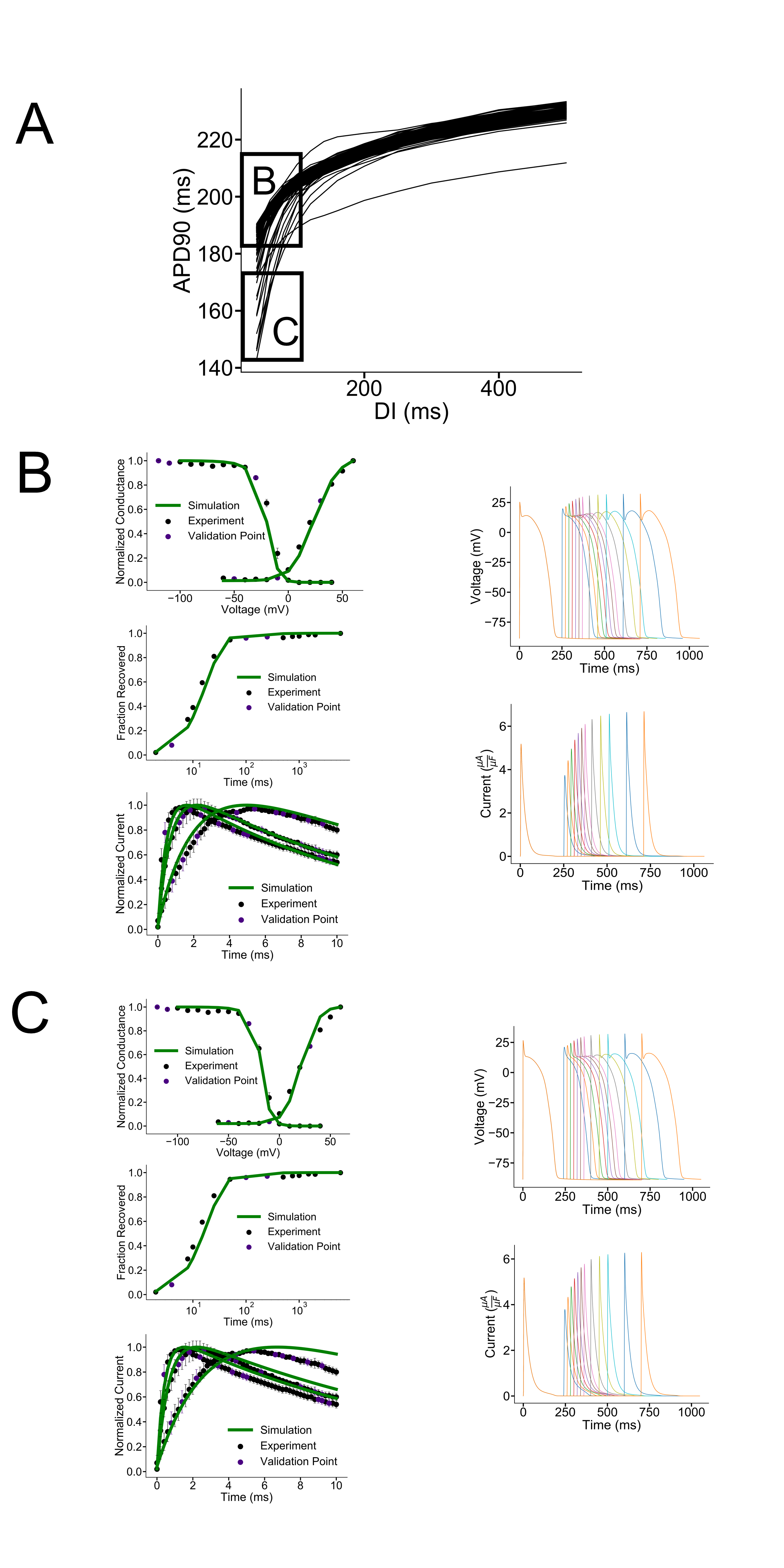

Supplement: S10 Fig — A) Boxes Labeled B and C correspond to the regions of the restitution curve of shallower B) and steeper C), respectively. B) Left: Acceptable model voltage protocol fits. Right: Voltage and Ito,f traces following the 10th S1 stimulus followed by a S2 stimulus at various DI intervals. Ito,f inactivates completely in 100 ms, resulting in a shallower restitution slope between 60–80 ms DI intervals. C) Left: Another acceptable model voltage protocol fits. Right: Voltage and Ito,f traces during the 10th S1 stimulus followed by a S2 stimulus at various DI intervals. In this acceptable model, Ito,f does not inactivate until 200 ms, resulting in a steeper restitution slope between DI intervals 60–80 ms. (TIF) [file pcbi.1008932.s011.tif]

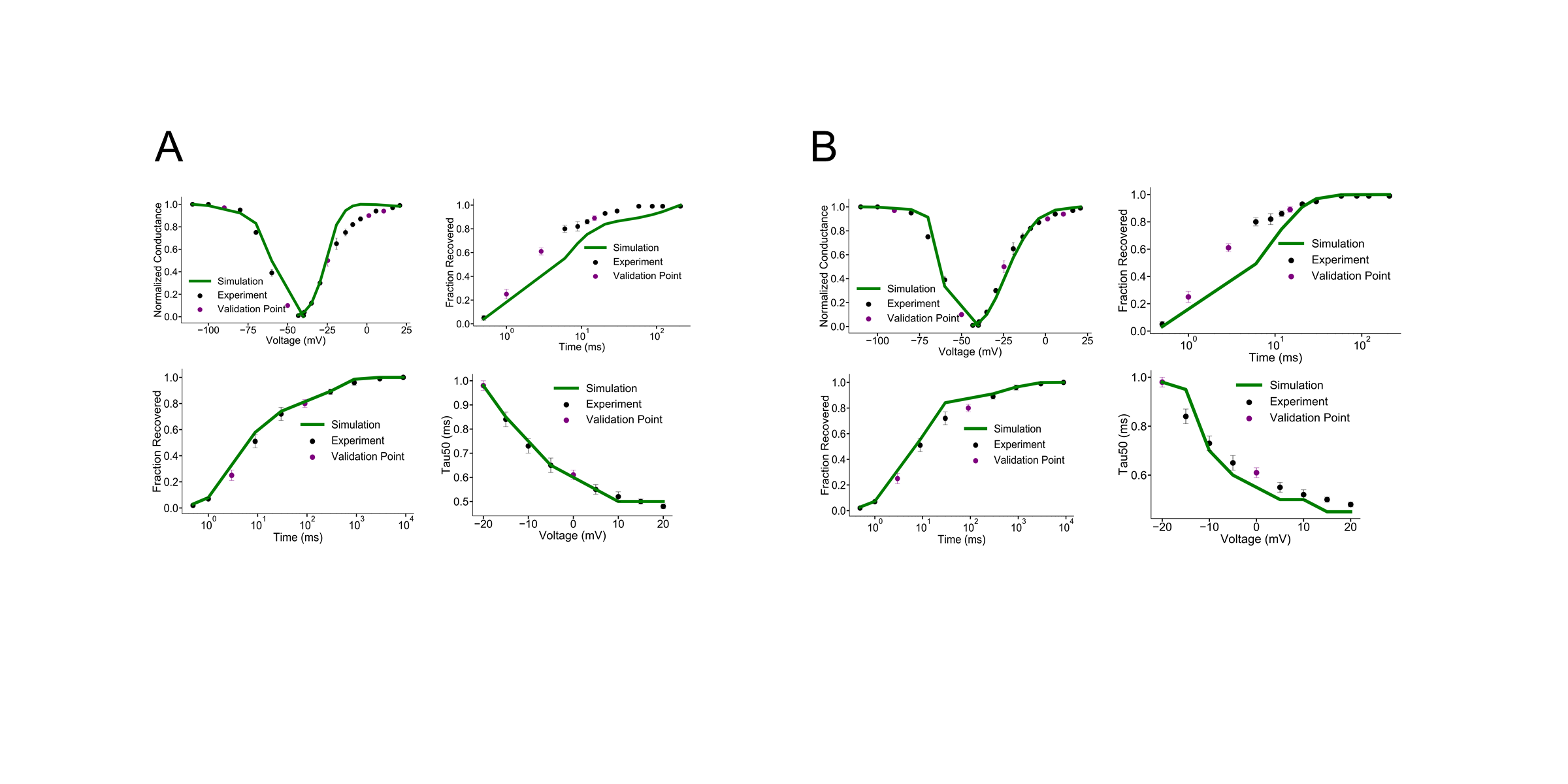

Supplement: S11 Fig — Both representative models show acceptable INa HEK voltage-clamp fits but the activation timing differs: 4 ms (A) versus 8 ms (B) (See Fig 5B). (TIF) [file pcbi.1008932.s012.tif]

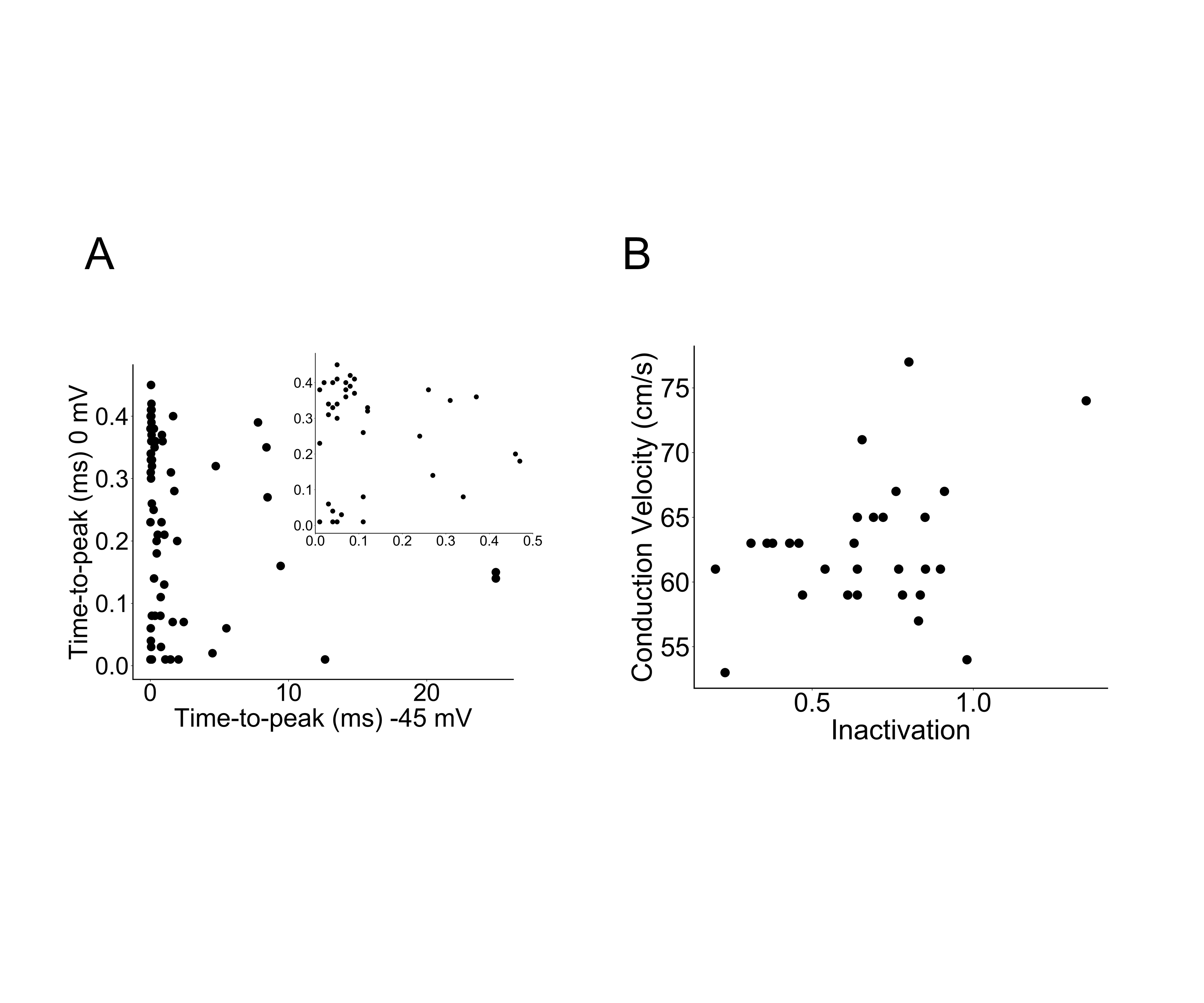

Supplement: S12 Fig — A) Time to peak at two voltages -45 mV and 0 mV starting from a holding potential of -86.2 mV as in ten Tusscher [52]. Inset: Zoomed in version of A between 0–0.5 ms. B) Conduction velocity vs inactivation of acceptable INa models with time to peak between 0.07–1.00 ms at both -45 mV and 0 mV to control for the extent of activation. Inactivation is the maximum open probability ratio at -20 mV for 10 ms following two conditions: 1) -90 mV at steady state, 1000 ms at 40 mV, -90 mV for 10 ms 2) hold at -90 mV. (TIF) [file pcbi.1008932.s013.tif]
