# Supplementary material for: Identification of structures for ion channel kinetic models
Source: PLoS Comput Biol. 2021 Aug 16;17(8):e1008932. doi: 10.1371/journal.pcbi.1008932 (PMC8389848; doi:10.1371/journal.pcbi.1008932)
Supplement: Attachment — Submitted filename: PLOS_COMP_BIO Reveiwer Responses Revision.docx [file pcbi.1008932.s014.docx]

**Responses to reviewers’ comments:**

Reviewer #1:

In this study by Mangold and colleagues, the authors demonstrate a generalized approach to identify acceptable Markov chain models representing ion channels kinetics. As the authors note, this is a critical challenge in the electrophysiology field, as most modeling approaches consider fits of rate constant parameters but provide much less consideration of the Markov chain model “structure” or “topology.” The authors systematically identify model topologies with reasonable biophysical constraints and determine acceptable and unacceptable fits to ion channel data for two different ion channels.

Overall, I find the study to be rigorously performed and the manuscript to be well written. I have a few specific comments for the authors to address.

**We thank this reviewer for the positive feedback and the helpful comments.**

Major:

1. The most significant issue that I see is that many of the general conclusions given regarding Markov chain topology and acceptable/unacceptable fits are described qualitatively. However, the authors have the data to provide quantitative measurements. (There are some specific numbers in the Discussion provided but not more generally for all cases.)

Specifically, as an example, the dots in Figure 4 provide a general sense of how the number of states and edges and root degree impact acceptable, tentative, and unacceptable fits. Please provide some quantitative measurements of this data, e.g., how does the percentage of acceptable fits change as the network properties changes?

**Thank you for this comment and encouraging us to provide more quantitative analysis. We have amended the results to include quantitative statements to guide the reader. Please see lines 300-310 and 321-326.**

2. The authors illustrate nicely the differences in acceptable vs unacceptable fits, in terms of fitting the data and also matching action potential characteristics. However, it is less clear how different or similar multiple “acceptable” fits are, specifically when incorporated into cellular and/or tissue models and tested outside the conditions in which the fits are performed.

For example, for the Ito,f current, the authors illustrate the fit to a specific S1S2 pacing protocol with a 200 ms S2 interval. If the authors measured a dynamic APD restitution curve with different Ito,f models in a ventricular cell model, how much variation would there be between different “acceptable” fits? Do the differences depend on whether or not the model is a more complex or simpler model?

For the INa current, for example, are there differences in conduction velocity (CV) and/or CV restitution for different “acceptable” fits in a 1D tissue, and if so, is there an explanation for these differences?

More generally, are “acceptable” fits for the same network topology more similar, compared with fits from different topology?

The authors address some of these issues in the Discussion, noting that different “acceptable” fits are practical for different situations, and I appreciate that answering the above questions for all “acceptable” fits may not be a computationally feasible exercise, in particular for the tissue simulations, but providing some examples would greatly strengthen the paper.

**Thank you very much for the comment and suggestion. We have added figures of acceptable I_to,f_ models in a dynamic restitution protocol and conduction velocities of acceptable I_Na_ models in a cable (Figure 5). There is considerable variability in the restitution curve slopes (Figure 5A) and in the conduction velocities (Figure 5B) amongst the acceptable models. It does not follow model complexity; rather it is a problem of parameter identifiability/lack of current traces in the voltage protocols. We have added additional supplemental figures (S10-S12) illustrating models with acceptable voltage protocol fits but with variation when simulating current in cellular and tissue dynamics.**

**We agree that this is a very nice way to illustrate the need for improved voltage protocols that reliably constrain these action potential current features. Please see lines 331 -354 and 447-454 for the presentation and discussion of these results.**

Minor:

1. There is a grammatical issue with the sentence that starts at line 65. The authors likely mean “Both cases…” instead of “In both cases”

**We have corrected this typo, thank you.**

2. Lines 391-2: The authors state there 167 distinct topologies but only 169 acceptable model solutions. Is one of these numbers a typo?

**Those numbers are correct. The numbers reflect the fact that the I_Na_ dataset is much harder to fit compared to the I_to,f_ dataset, for which there is a wealth of acceptable solutions (11 topologies with 156 acceptable solutions). Please see lines 467-472.**

3. In several of the figures, a red asterisk is placed next to some of the network topologies. What does this represent? Please include this detail in the figure caption.

**We apologize for including those red asterisks from previous paper drafts by mistake. They have been removed.**

4. Some aspects of the optimization procedure are difficult to follow, as described in the Results and Methods. In particular, I did not understand the explanation of progress, generalization loss, and Q as shown in Figure S2.

**We have added clarifying text to the Methods (lines 564-568) and updated Figure S2** **and legend.**

Reviewer #2: In the paper by Mangold and co-authors, the authors set out to develop a systematic approach to identify topologies for Markov model representations of ion channels. This is a great paper. The authors demonstrate a potential solution to a long-standing problem: how to identify the appropriate Markov state model that best represents ion channel gating for a particular ion channel species.

One aspect of this paper that is particularly innovative, is that the authors utilize biophysical properties of ion channels to restrict the number of available topologies. This is an advantage over standard combinatorial methods, which generally results in large numbers of models that have no physiological basis in reality.

I really didn’t find anything not to like in this paper. I think the methodology is useful, will be widely adopted, and it’s a starting point that will allow for continued improvement. Figures are clear and the writing is excellent and manages to take a complex topic and make it accessible.

My major comment is that I hope the source code utilized in the paper can be made freely available. I didn’t see a link to the source code on the github, but would ask that the authors make that available to allow the whole process to be utilized by others. User friendly will be the key here - please make sure the codes are well documented and user friendly.

**Thank you for your enthusiasm for our work/paper.**

**We apologize that you were unable to locate the link to the source code on Github as it was deposited there, and we have confirmed that it is available there** (**https://github.com/mangoldk/AdvIonChannelMMOptimizer.) We hope the reviewer (and others) will let us know if access problems remain.**

Reviewer #3:

This paper attempts an exhaustive enumeration and validation of multi-state kinetic models of voltage gated ion channels, evaluated for quality of fit to data for several specific channels from cardiac myocytes.

I am very sympathetic to the aims of this study. I think this is a timely and important problem. Robust and principled approaches to identifying ion channel models are needed, and in my view this study goes some way towards achieving this goal.

My main critique of the paper is in its presentation. With apologies to the authors for my bluntness, large parts of the paper read as a project progress report (... first we did this, next we did that....). The language is often quite informal and imprecise. There is frequent use of vague qualifiers, such as 'a reasonable number of ...', '...still quite large.' Such vague statements are not appropriate. Furthermore, there are statements made for which no justification is given. As an example, page 5, 'These rates would be increasingly difficult to identify experimentally as the number of connections increases.' No justification for this statement is seemingly provided in the text. This is one of many such instances. I suggest the authors reread their text and where such statements are made, provide justification or remove. Finally, large sections of the manuscript have no subheadings to help guide the reader, and were hard to navigate and in all honesty rather monotonous as a consequence: in particular the Results section and the Discussion and Future Directions section. I would strongly encourage the authors to clarify what are the major points that they wish to get across, and shorten and subdivide / subtitle these sections accordingly.

**We are grateful for your honest assessment and helpful suggestions. In revising the manuscript, we have made every effort to remove vague and qualitative language and to replace it with quantitative language whenever and wherever possible. In addition, we have added section headers in both the Results and Discussion sections of the manuscript.**

There is also a problem with the current order of the paper, in that some concepts that are in my view critical to understanding what the authors are doing are presented towards the end in the method section. In particular, the actual model being used (the form of the rate constants including voltage dependence, for example) doesn't get discussed until then. In order for the presentation to be intelligible these concepts (but not specific implementation details) need to come earlier (perhaps even in the introduction, given that they were taken from earlier work).

**Thank you for this excellent suggestion. We have moved discussion of the rate parameters to an early section in the Results, starting at line 143.**

Some questions that may be of interest to address: I have never understood why ion channel models only consider one open state. Is there a good reason for this? If so it would be helpful to mention it.

**Thank you for raising this excellent point. There is no *a priori* reason why channel gating models cannot include more than open state. Indeed, we are aware of experimental evidence for multiple open states in some types of ion channels, including KCNQ1- and hERG-encoded potassium channels, suggesting that including multiple open states will be needed for some channels. We present datasets here with a single open state for simplicity, but our routine could certainly be amended to include more than one open state. (The isomorphism testing routine handles multiple open states if the researcher needs that additional complexity.) We have added language in the Discussion and in the Methods sections of the manuscript to address this question directly. (Lines 485,499-500)**

Cycles are highly problematic in this type of model unless adequately dealt with, for two reasons. Firstly equilibrium constraints mean that for each and every cycle there is a parameter dependence that has to be satisfied (I think the authors mention this, again not until the methods - this needs to be mentioned earlier). Previous authors have also found that even when these constraints are made explicit, parameter identification with cycles is problematic (presumably because of difficulties with unique parameter identification). Did the authors observe any differences between models with / without cycles when fitting to data?

**Yes, absolutely you are correct: each cycle introduces one dependent rate constant. As suggested, we have moved our rate constant formulation to the beginning the manuscript. For the less complex macroscopic dataset, I_to,f,_ possible structures are both linear and cyclic. However, the more complex I_Na_ dataset clearly favors models with cycles. This difference reflects the fast excitation cycle of I_Na_, more specifically the fast activation and inactivation of I_Na_, followed by relatively slow deactivation. Having a cycle between these various states allows for the necessary flow of state probability without having to worry about bursting back through the open state, as is evident in a sparsely connected model. Please see lines 414-418.**

The authors do not give much attention to the question as to the data needed to identify models. Effectively what the approach outlined in this manuscript will ideally determine is what is the best model given the data. This of course raises the issue as to whether other models might actually be better if more data or different data were available. I wonder whether the authors have any thoughts on this other dimension to the model identification challenge here?

**This is another excellent point. We agree completely and we thank you for encouraging us to reflect on this challenge. As you mentioned, our approach only identifies the best collections of models given an experimental dataset. With additional data we may very well see that different topologies better represent that expanded dataset. We hope that our user-friendly routine will be run multiple times as more data becomes available so it will be possible to begin to understand what topological features are important to model specific aspects of each experimental dataset. We have added language in the Discussion (lines 421-425) to address this important point.**

I was confused by Figure 1A. It isn't clear to me whether this is trying to illustrate the unique topologies with three nodes, or trying to illustrate different ways in which topologies may be considered repeats in respect of ion channel models. On the top line the first and second models are clearly different, but the third is a relabled version of the first. How is this indicated through the highlighting was not clear to me, especially as further down in the first line with cyclic models, the two pink highlighted models now do appear to be the same.

**Thank you for pointing out the confusion with the presentation of Figure 1A.To clarify, we have amended Figure 1A and the figure caption to clarify. In Figure 1A, we are showing all the possible ways 3-state rooted graphs can be labelled. Of the 36 permutations, 6 belong to the original rooted topologies (outlined in black boxes). Three of these six are isomorphic (highlighted with orange shading) while the other three are unique (no shading), as also depicted in Figure 1B. The 30 topologies highlighted yellow in Figure 1A are the remaining permutations.**

Finally, are there any insights that can be gained into the biology of ion channels that arise from trying to relate the topologies that are seemingly preferred in this methodology to known biophysical characteristics of ion channel proteins? In particular this question is raised because, perhaps naively, I would have expected models with topologies similar to those shown in Figure 3B to be more the commonly accepted topologies (including as determined in for single channel data), and would have thought models with more cycles as in Figure 3A, to be less representative of current modelling approaches.

**As noted in our response above, topologies with cycles tended to reproduce macroscopic I_Na_ successfully. It appears that this trend reflects the fast excitation cycle of I_Na_, that is, fast activation and inactivation followed by relatively slow deactivation. Having a cycle between these various states allows for the necessary flow of state probability without having to worry about bursting back through the open state in a sparsely connected model.**
